# Novel therapies for mucopolysaccharidosis type III

**DOI:** 10.1002/jimd.12316

**Published:** 2020-09-28

**Authors:** Berna Seker Yilmaz, James Davison, Simon A. Jones, Julien Baruteau

**Affiliations:** ^1^ Genetics and Genomic Medicine, Great Ormond Street Institute of Child Health University College London London UK; ^2^ Department of Paediatric Metabolic Medicine Mersin University Mersin Turkey; ^3^ Metabolic Medicine Department Great Ormond Street Hospital for Children NHS Foundation Trust London UK; ^4^ Metabolic Medicine Manchester University NHS Foundation Trust Manchester UK; ^5^ National Institute of Health Research Great Ormond Street Hospital Biomedical Research Centre London UK

**Keywords:** adeno‐associated virus, enzyme replacement therapy, gene editing, gene therapy, heparan sulfate, lentivirus, lysosomal storage disease, mRNA, mucopolysaccharidosis type III, Sanfilippo disease, substrate reduction therapy

## Abstract

Mucopolysaccharidosis type III (MPS III) or Sanfilippo disease is an orphan inherited lysosomal storage disease and one of the most common MPS subtypes. The classical presentation is an infantile‐onset neurodegenerative disease characterised by intellectual regression, behavioural and sleep disturbances, loss of ambulation, and early death. Unlike other MPS, no disease‐modifying therapy has yet been approved. Here, we review the numerous approaches of curative therapy developed for MPS III from historical ineffective haematopoietic stem cell transplantation and substrate reduction therapy to the promising ongoing clinical trials based on enzyme replacement therapy or adeno‐associated or lentiviral vectors mediated gene therapy. Preclinical studies are presented alongside the most recent translational first‐in‐man trials. In addition, we present experimental research with preclinical mRNA and gene editing strategies. Lessons from animal studies and clinical trials have highlighted the importance of an early therapy before extensive neuronal loss. A disease‐modifying therapy for MPS III will undoubtedly mandate development of new strategies for early diagnosis.

## 1 INTRODUCTION

Mucopolysaccharidosis type III (MPS III, Sanfilippo syndrome) is a progressive neurodegenerative lysosomal storage disorder (LSD), caused by biallelic mutations in one of four genes encoding enzymes involved in the degradation of heparan sulfate (HS).^1^ While the central nervous system degeneration is the predominant feature, progressive multisystem somatic disease is also seen although to a lesser extent than in MPS I (Hurler disease) or MPS II (Hunter disease). MPS III has a prevalence of 1:50 000 to 1:250 000, with MPS IIIA and IIIB being the commonest forms, and MPS IIIC and IIID being much rarer (Table 1).

**TABLE 1:** Characteristics of MPS III subtypes

	OMIM	Enzyme	Gene	Proportion of MPS III patients (from Reference 2)	Geographic (from Reference 1)	Common variants
MPS IIIA	#252900	N‐sulfoglucosamine sulfohydrolase (sulfamidase, heparan sulfate sulfatase)	*SGSH* (605270)	60%	Northern/Eastern European. Cayman islands (from Reference 7)	c.734G > A c.220C > T c.1139A > G c.197C > G (from Reference 1)
MPS IIIB	#252920	N‐acetylglucosaminidase alpha	*NAGLU* (609701)	30%	Southern European	c.889C > T c.1000G > T (from Reference 1)
MPS IIIC	#252930	Heparan‐alpha‐glucosaminide N‐acetyltransferase	*HGSNAT* (610453)	4%		c.1030C > T (from Reference 10)
MPS IIID	#252940	N‐acetylglucosamine‐6‐sulfatase (glucosamine‐6‐sulfatase)	*GNS* (607664)	6%	Italian, Turkish (from Reference 11)	

Abbreviation: MPS III, mucopolysaccharidosis type III.

### 1.1 Natural history

**FIGURE 1:**
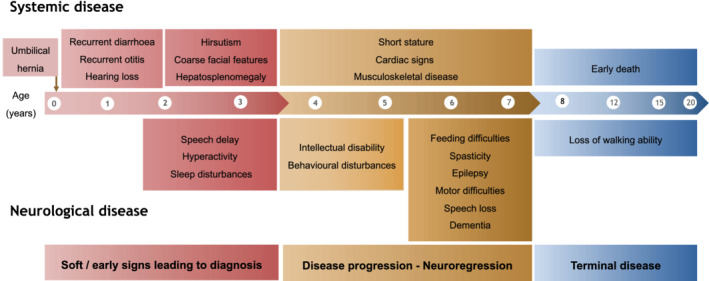
Neurovisceral disease in mucopolysaccharidosis type III. This schematic representation of the classical form of the disease show the main symptoms with age of onset

Common initial presenting features in patients with MPS III include developmental delay, speech delay, recurrent ear/nose/throat infections, and in some patients diarrhoea, or hearing loss.^2^ A typical progression through three phases has been described, with initial developmental delay from 1 to 4 years of age, often with recurrent ear infections. At an early stage, patients can be misdiagnosed with idiopathic developmental delay, attention deficit/hyperactivity disorder or autism spectrum disorder.^3^ This is followed from 3 to 4 years of age by severe behavioural disturbance, and in many patients subsequent development of seizure disorders. Sleep disturbance is frequent and causes sleep onset latency, frequent night time waking, and daytime sleep.^4^ Cardiac signs are often reported, which includes a valvular disease and occasionally left ventricular hypertrophy. Osteopenia caused by immobility or vitamin D shortage potentially complicated by pathological fractures, scoliosis, mild‐to‐moderate joint stiffness, carpal tunnel syndrome, and avascular necrosis of the femoral head represent a common musculoskeletal presentation of the disease.^5^, ^6^ From 10 years onward, there is a further phase of deterioration with increasing falls and immobility, feeding, and swallowing impairment necessitating enteral tube feed support, increasing spasticity, and expected mortality during adolescent years usually due to recurrent respiratory infections (Figure 1).^2^, ^7^ Aside from this severe and rapidly progressing phenotype, some patients might present with a more slowly progressing phenotype.^8^ A cohort of adult MPS IIIA patients was recently described with retinal dystrophy and hypertrophic cardiomyopathy, while only 25% of patients had neurocognitive decline.^9^ In comparison to MPS IIIA and IIIB, MPS IIIC and IIID have a slightly milder course with a longer survival compared to the other two types.^10^, ^11^, ^12^

More detailed natural history studies have been recently published for MPS IIIA^8^ and MPS IIIB,^13^ detailing the expected cognitive trajectory over time periods relevant for potential interventional clinical trials.

### 1.2 Pathophysiology

**FIGURE 2:**
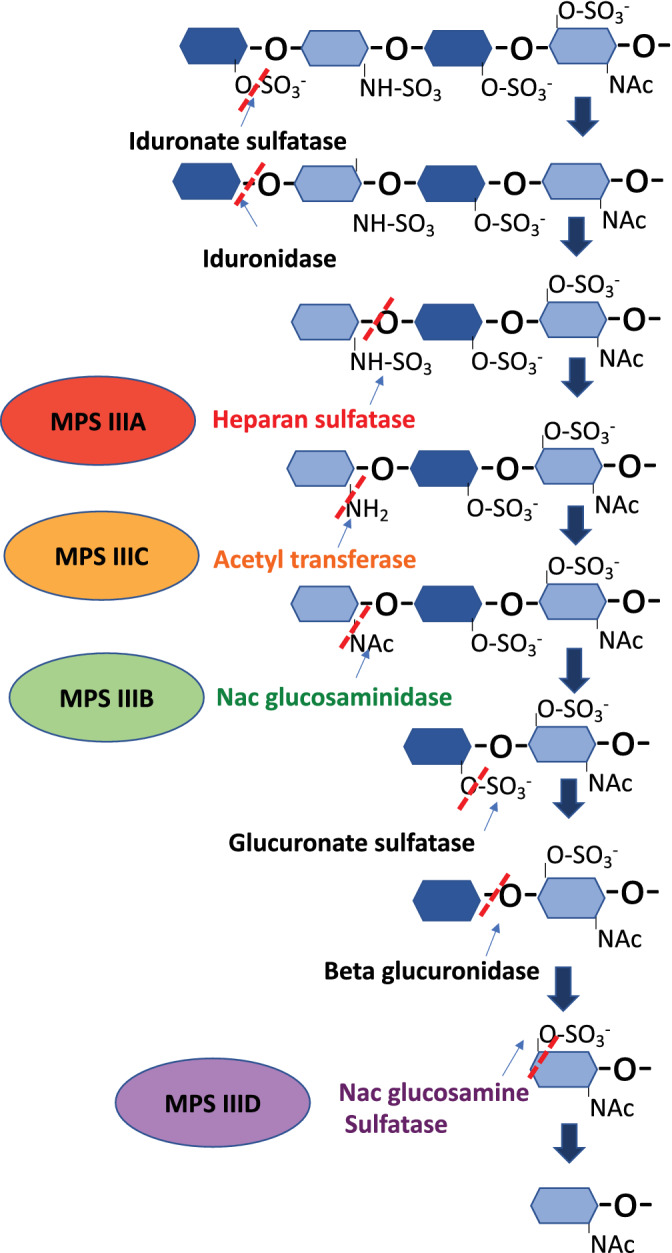
Degradation of the glycosaminoglycan chain heparan sulfate. The deficient enzymes involved in the different subtypes of MPS type III are highlighted. MPS III, mucopolysaccharidosis type III; NAc, N‐acetyl; SO3−, sulfate

HS is a polysaccharide composed of repeated disaccharides including the most common moiety, glucuronic acid linked with N‐acetylglucosamine. HS is degraded within the lysosomal system by a sequence of lysosomal hydrolases, commencing with iduronate sulfatase (deficient in MPS II), then iduronidase (deficient in MPS I), followed by heparan sulfatase (MPS IIIA), acetyl‐transferase (MPS IIIC), N‐acetyl‐glucosaminidase (MPS IIIB), and further distally N‐acetyl glucosamine sulfatase (MPS IIID) (Figure 2).^14^

In patients with MPS III, there is mainly accumulation of HS which can be detected in the urinary glycosaminoglycans assay. The classical view of the pathophysiology was of lysosomal storage of HS, resulting in organelle‐level, cellular‐level, and finally tissue‐level dysfunction. It is now known that there is also secondary storage of other products due to the lysosomal disruption, including gangliosides, cholesterol, ceramides, and sphingomyelin.^15^

Recent studies have shed new light on MPS III neuropathology. Neuroinflammation is a hallmark of MPS III neurological disease with massive astrocytic and microglial activation.^16^ Both innate and adaptive immune responses are involved with dysregulation of macrophages, B‐cells, T‐cells, cytokines, and complement factors^17^ and can partially respond to anti‐inflammatory therapy.^18^, ^19^ HS, which binds Toll‐like receptor 4^20^ and mediates microglial activation, may be the principal trigger although additional storage molecules like GM2 and GM3 molecules^21^, ^22^ or an autoimmune component^23^, ^24^ have been suggested. The extracellular accumulation of HS also interacts with adhesion molecules contributing to microglial activation.^25^, ^26^

Lysosome are essential organelles for clearance of macromolecules but play a role in regulation of cellular processes, for example, cell cycle, cytoskeleton and intracellular transport, endoplasmic reticulum and Golgi functions. Some recent studies suggest that MPS III lysosomes could dysregulate key cellular functions such apoptosis and autophagy.^27^, ^28^ Impaired autophagy seems involved in accumulation of aggregation‐prone proteins and subsequently elevated amyloid‐β, tau, and α‐synuclein, which are well‐known biomarkers of neurodegeneration.^17^ Mitochondrial dysfunction with impaired mitophagy has also been described. HS plays physiological roles in neurogenesis, axonal guidance, and synaptogenesis^29^ and its accumulation will disrupt these mechanisms. Abnormal membrane composition has been observed in MPS III cells, which impairs vacuolar transport, synaptic vesicular recycling, and neurotransmitter release.^30^

The overall resultant effect is of progressive neurodegeneration, which then manifests with the clinical phenotype described.

### 1.3 Diagnosis

If there is clinical suspicion of MPS disorder, the first screening test is to assay the urinary glycosaminoglycans which can be quantified, and then if elevated individual GAG species identified by either electrophoretic or mass spectroscopy methods. Identification of prominently elevated HS will be followed by enzyme activity assay in leucocytes to determine MPS III subtype.^14^ Molecular genetic analysis will confirm biallelic mutations and may provide prognostic information (see below). In some situations, the diagnosis is initially reached by molecular genetic analysis, for example via whole exome sequencing,^9^ which will then be substantiated by biochemical methods to confirm pathogenicity of the genetic variant(s).

### 1.4 Genotype‐phenotype correlation

A genotype‐phenotype correlation has been identified for some gene mutations in MPS IIIA and IIIB,^31^, ^32^ but not for IIIC/D. More deleterious mutations (eg, premature termination codons, nonsense mutations) are associated with more rapidly progressive disease, while less deleterious missense mutations have been associated with attenuated phenotypes. The clinical phenotype will depend on the severity of the combination of biallelic variants.

### 1.5 Standard of care

Until now, no effective disease‐modifying treatment has been identified, and supportive treatment to address the various multisystem problems is the mainstay of therapy. This requires a multidisciplinary approach to management, anticipating the likely progressive problems that will arise over time.

## 2 HAEMATOPOIETIC STEM CELL TRANSPLANTATION FAILURE

In early stages of some neurological LSDs, haematopoietic stem cell transplantation (HSCT) may stop neurological disease progressions, including MPS I^33^, ^34^ and metachromatic leukodystrophy.^35^, ^36^ However, HSCT has not provided any stabilisation or improvement in cognitive function in MPS III patients,^37^, ^38^ even if the procedure is performed before 2 years of age.^39^ Wild‐type donor cell transplant (WT‐HSCT) decreased GM2 ganglioside storage and neuroinflammation in MPS IIIA mice, but were less effective at reducing HS and had no significant effect on behaviour.^40^ HSCT performed in adult MPS IIIA mice showed a mild decrease of cerebral storage material which did not translate into phenotypic improvement.^41^ Neonatal HSCT in MPS IIIA mice neither achieved increased N‐sulfoglycosamine sulfohydrolase (SGSH) activity in visceral organs and the brain, nor improved HS storage and neuropathology. An insufficient donor‐derived enzyme production and/or uptake by host cerebral cells was hypothesised.^42^ Similarly, HSCT did not show any benefit on lifespan, motor function, or central nervous system (CNS) lysosomal storage in neonatal MPS IIIB mice.^43^

## 3 ENZYME REPLACEMENT THERAPY

Enzyme replacement therapy (ERT) provides a recombinant functional enzyme to deficient cells via the mannose 6 phosphate (M6P) receptor endocytosis pathway, which targets extracellular M6P‐tagged proteins to the lysosome. ERT has become the standard of care in several LSDs,^44^ especially in MPS type I, II, IVA, VI, and VII. Systemic ERT has shown improvement in joint mobility and walking ability, respiratory function, reduction of liver, and spleen volumes concomitant with decrease in urinary GAG (uGAG) excretion.^45^, ^46^, ^47^, ^48^, ^49^, ^50^, ^51^, ^52^, ^53^, ^54^, ^55^ Systemic ERT has limited ability to cross the blood‐brain barrier (BBB)^56^ and does not modify the neurological phenotype.^57^ Other organs with limited blood supply or slowly dividing tissues like bone, cartilage, heart valves, or the eye display limited benefit from ERT.

**FIGURE 3:**
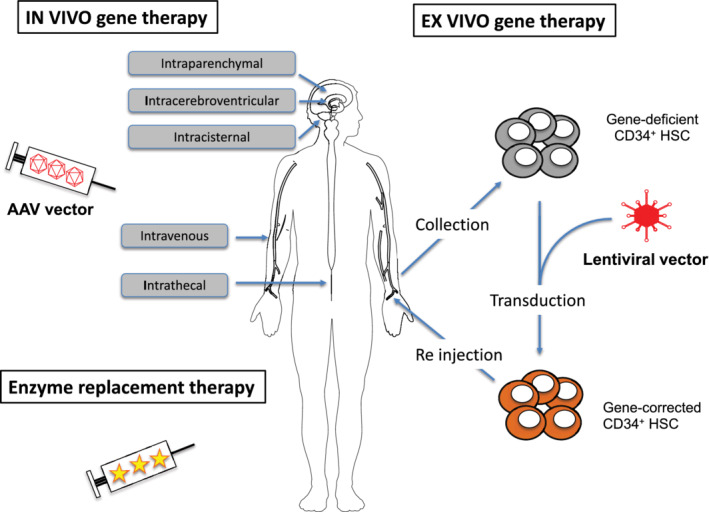
General principles and main routes of administration of enzyme replacement and adeno‐associated viral (AAV) and lentiviral vectors mediated gene therapy. HSC, haematopoietic stem cell

In MPSIII, neurodegeneration is the main debilitating clinical sign requiring efficient CNS targeting.^56^, ^58^ Alternatives to IV administration have been proposed such as direct intraparenchymal (IP), intrathecal (IT), or intracerebroventricular (ICV) administration (Figure 3).^59^ Although delivered centrally, the ability to deliver ERT uniformly to diffuse cerebral regions after IT or ICV routes of administration is not fully clarified. ERT trials are summarised in Table 2.

**TABLE 2:** Trials of ERT for MPS III

	Investigational medicinal product	Sponsor	Administration route	Study status	Dose	Clinical trial identifier
MPS IIIA	rhSGSH	Shire	IT (drug delivery device)	Completed	10 mg, 45 mg, 90 mg monthly	NCT01155778
				Terminated		NCT01299727
	rhSGSH (HGT‐1410)	Shire	IT (drug delivery device)	Completed	45 mg Q2W, 45 mg Q4W, placebo	NCT02060526
				Terminated		NCT02350816
	rhSGSH (SOBI003)	SOBI	Intravenous	Completed	3 mg/kg, 10 mg/kg, and a higher dose decided weekly	NCT03423186
				Active, not recruiting	Will be determined later	NCT03811028
MPS IIIB	rhNAGLU‐IGF2 (tralesinidase alfa)	Allievex Corporation	ICV	Active, not recruiting	30 mg, 100 mg, 300 mg weekly	NCT02754076
				Enrolling by invitation	300 mg weekly	NCT03784287
	rhNAGLU (SBC‐103)	Alexion	Intravenous	Terminated	1 mg/kg QOW, 3 mg/kg QOW	NCT02618512

Abbreviations: ERT, enzyme replacement therapy; ICV, intracerebroventricular; IGF2, insulin‐like growth factor 2; IT, intrathecal; MPS III, mucopolysaccharidosis type III; rhNAGLU, recombinant human α‐N‐acetylglucosaminidase; rhSGSH, recombinant human sulfamidase; SOBI, Swedish Orphan Biovitrum.

### 3.1 MPS IIIA

Following successful IT ERT in MPS I dogs,^60^ MPS II mice^61^, ^62^ and MPS VI cats,^63^, ^64^ MPS IIIA mice received recombinant human sulfamidase (rhSGSH) via direct injection in the cisterna magna, which showed a fall of cerebral and medullar HS levels and improved behaviour.^65^ Subsequently, intracisternal (ICM) ERT in MPS IIIA Huntaway dogs confirmed an increase up to 39‐fold of normal cerebral SGSH activity with decreased cerebral HS levels^66^ and remarkable improvement in both neuronal storage and inflammation in presymptomatic MPS IIIA dogs.^67^ Moreover, intermittent cisternal or spinal bolus injection of rhSGSH provided greater reductions in substrate storage and neuroinflammation than slow continual spinal enzyme infusion in MPS IIIA dogs.^68^ In MPS IIIA mice, ICV and ICM routes of administration were also found more effective in decreasing substrate levels and reducing microglial activation than IT.^69^ Safety of rhSGSH was successfully assessed in juvenile Cynomolgus monkeys via an IT drug delivery device (IDDD)^70^ and in MPS IIIA dogs after IT injection.^71^ These promising preclinical studies paved the way to early phase clinical trials.

A Shire‐sponsored open‐label, phase I/II, safety trial of IT rhSGSH via IDDD (NCT01155778) recruited 12 patients aged 3 years and above receiving monthly administration for 6 months in escalating doses. No safety concern of rhSGSH was observed, but seven patients experienced serious adverse effects with all but one related to non‐functioning IDDD, that is, migration, disconnection or pinbreak. Although plasma anti‐rhSGSH antibodies were detected in six patients, CSF HS and uGAG levels were reduced in all tested patients in a dose‐response pattern. Neurocognitive assessments showed a decline in four patients and a stabilisation in six patients (no data available in two patients) with no dose difference. Brain MRIs showed a worsening cortical atrophy in all dose groups although this 6‐month duration study was too short to adequately assess clinical efficacy.^72^ Patients were subsequently recruited in an open‐label extension study (NCT01299727) for the initial established dose; however, the study was terminated as pre‐specified efficacy criteria were not met.

Furthermore, a 48‐week, phase IIb, open‐label, randomised, safety and efficacy study of rhSGSH administration via an IDDD was initiated in early stage paediatric MPS IIIA patients in 2014 (NCT02060526).^73^ Twenty‐one patients (12 females and 9 males) with a mean age of 32 months were randomly divided into three groups: IT administration of 45 mg rhSGSH every 2 weeks (Q2W), IT administration of 45 mg rhSGSH every 4 weeks (Q4W), or no treatment. The primary endpoint was set as maximum 10‐points decline of DQ after 48 weeks. A satisfactory safety profile of rhSGSH was observed and all serious SAEs were related to IDDD. A clinical response to rhSGSH was observed only in three treated patients (two in the Q2W group, one in the Q4W group) with no significant difference between treatment and control groups. Contrasting with reduction of CSF HS and uGAG levels in all treated patients, efficacy endpoints were not met and the trial terminated in 2016.^73^

A Swedish Orphan Biovitrum (SOBI)‐sponsored open‐label, phase I/II study (NCT03423186) is ongoing based on weekly IV administration of SOBI003 in 3 dose‐escalating cohorts a chemically modified rhSGSH for 24 weeks. A glycan modification of rhSGSH using the proprietary technology Modifa is claimed to extend the half‐life of the enzyme.^74^ The primary objective is to assess the safety of SOBI003. Secondary outcomes focus on pharmacokinetics, immunogenicity, and efficacy based on neurocognition, behaviour, neuroimaging, and quality of life changes. Patients in the first dose group showed good tolerability after completing 24 weeks infusions. An extension study (NCT03811028) is set up for the further 80 weeks.^75^

### 3.2 MPS IIIB

Recombinant human α‐N‐acetyl glucosaminidase (rhNAGLU) has reduced cellular uptake due to limited cation‐independent mannose 6 phosphorylation.^56^, ^76^, ^77^ As the M6P receptor is also the receptor for IGF2 at a different binding site,^78^ the rhNAGLU enzyme was fused to the receptor‐binding motif of IGF2 (insulin‐like growth factor 2) (rhNAGLU‐IGF2) to improve its cellular uptake. Preliminary in vitro work has suggested that this rhNAGLU‐IGF2 fusion might improve neuronal and astrocytic targeting.^79^

A Biomarin‐sponsored open‐label, phase I/II dose‐escalation study to evaluate the safety and efficacy of BMN‐250 (rhNAGLU‐IGF2) in MPS IIIB patients (NCT02754076; long‐term extension study NCT03784287) started in 2016 with an initial dose escalation (30, 100, and 300 mg/infusion) via ICV infusion weekly until reaching the maximum tolerated tested dose, which was then administered for 48 months. BMN‐250 or tralesinidase alfa is a development programme out‐licensed to Allievex Corporation in 2019. Tralesinidase alfa was well tolerated and CSF HS levels came into a normal range in all seven treated patients within 1 to 3 weeks of treatment. After 24 weeks of treatment, liver and spleen volumes reached normal range in 9/9 and 7/9 subjects, respectively. DQ were stabilised in 5/7 treated patients.^80^ The extension study (NCT03784287) was started in 2018 and weekly ICV administration of 300 mg dose will continue up to 240 weeks.

An Alexion‐sponsored open‐label, phase I/II study (NCT02618512) based on IV administration of rhNAGLU (SBC‐103) was early terminated and only safety was evaluated in three recruited patients.^81^

## 4 SMALL MOLECULE THERAPY

Small molecule therapies act through various mechanisms such as read‐through inducers, pharmacological chaperones, proteostasis regulators, substrate reduction therapy (SRT), or autophagy inducers.^82^ These molecules are attractive due to their small size (usually <500 Da), which allow them to rapidly diffuse into cells, and cross the BBB. Therapeutic efficacy has been demonstrated on the neurological disease in some LSDs such as Niemann‐Pick disease type C, Fabry disease, Gaucher disease, and Mucolipidosis type IV.^83^ Oral intake is the usual route of administration.^83^

### 4.1 Substrate reduction therapy

SRT aims to decrease the upstream biosynthesis of the substrate of the deficient enzyme and so decrease toxic accumulation.^84^, ^85^ Considering the limited effects of ERT on several tissues and difficulties of BBB crossing, SRTs have emerged as attractive alternatives for LSDs, especially neurodegenerative diseases.^85^

Rhodamine B, an inhibitor of polysaccharide chain formation in GAG synthesis,^86^ is a small molecule of 479 Da which has the physical property to cross the BBB.^87^ Rhodamine B was effective in clearing GAG storage in MPS IIIA mice showing liver size reduction, decrease of GAG levels in the brain and somatic tissues.^88^ A 6 months therapy displayed improved learning and spatial memory.^89^ A trans‐generational study evaluating the continuous exposure to Rhodamine B in MPS IIIA mice over four generations (including pregnancies) did not report any adverse effects, confirmed reduction in liver size and GAG clearance with incremental efficacy over generations.^90^ The translation of these encouraging findings was hampered by toxic effects reported in the literature such as skin lacerations, gastrointestinal and liver tumours, and decreased fertility.^91^, ^92^, ^93^

Genistein, a protein‐tyrosine‐kinase inhibitor from the isoflavone group, inhibits GAG synthesis by inhibition of the epidermal growth factor receptor‐dependent signal, a concept described as “gene expression‐targeted isoflavone therapy” (GET IT).^94^ Although genistein has limited ability to cross the BBB with an estimated CNS delivery below 10%,^95^ in vitro proof of concept were observed in fibroblasts from MPS I, MPS II, MPS IIIA, and MPS IIIB patients.^96^ A synergistic effect was observed when combined to different isoflavones in human fibroblasts from MPS IIIA and VII patients.^97^ Genistein oral administration in MPS IIIB mice showed increased GAG clearance in the liver after 8 weeks^98^ and improved behaviour, neuropathology, and partial HS clearance after 9 months of daily administration at high dose (160 mg/kg/day).^99^

An open‐label pilot study was performed in a paediatric population of five MPS IIIA and five MPS IIIB patients with oral administration of genistein (5 mg/kg/day) for 12 months.^100^ Tolerance was satisfactory, uGAG levels decreased significantly in all MPS IIIA patients and in two MPS IIIB patients correlating with a significant improvement or stabilisation in cognitive functions.^100^ A 3‐year study with a genistein‐enriched preparation with multiple isoflavones (5 mg/kg/day) performed in six MPS IIIA and two MPS IIIB patients showed an initial improvement of cognitive functions in the first year in 7/8 patients, which was not sustained at 36 months.^101^ A randomised, crossover, placebo‐controlled study with a genistein‐enriched soy isoflavone extract (10 mg/kg/day of genistein) enrolled 30 MPS III patients for 6 months followed by an open‐label extension study for another 6 months for patients who were on genistein during the last part of the crossover.^102^ Neither clinical benefit nor uGAGs and HS reduction compared to placebo were observed at completion at 12 months.^102^ Higher doses of genistein in five MPSIIIA and one MPS IIIB infant patients with 15/mg/kg/day of genistein over 12 months^103^ suggested that higher dose could provide efficacy.^103^ An open‐label study enrolling 22 MPS II, III and VII patients with 150 mg/kg/day of pure genistein aglycone for 12 months was considered safe.^104^ Thus, a phase III, double blinded, randomised, placebo‐controlled clinical trial of high dose (160 mg/kg/day) oral genistein aglycone for 12 months then 12 months open‐label was initiated in 21 MPS III patients (subtypes A, B, and C) in 2014. Although there was a demonstrable reduction in urinary HS levels, the study revealed no significant CSF HS reduction and no clinical benefit from the neuropsychological tests performed.^105^

### 4.2 Pharmacological chaperone therapy

Chaperone therapy, or enzyme enhancement therapy, relies on small molecules, which inhibit the misfolding of mutant proteins preserving their active sites, increasing their stability and preventing aggregation.^106^, ^107^ Amino and imino sugars are the most common pharmacological chaperones used in LSDs including GM1‐gangliosidosis, Fabry, Morquio B, Pompe, Gaucher, Krabbe, Niemann‐Pick A/B, and C diseases.^108^ Orally administered chaperones can cross the BBB. However, the effect is mutation‐specific and can only benefit a small population of patients with these orphan diseases.^107^

Glucosamine, an aminosugar inhibiting competitively HGSNAT inhibitor, has shown in vitro proof of concept in fibroblasts from MPS IIIC patients with either missense^109^ or acceptor splice‐site mutations affecting the *HGSNAT* gene.^110^

### 4.3 Anti‐inflammatory therapy

As neuroinflammation is a hallmark of MPS III pathophysiology, the effect of anti‐inflammatory therapies has logically been considered.

Aspirin was tested in vivo in MPS IIIA mice at high dose (200 mg/kg) administered by intraperitoneal injection three times a week for 2, 4, or 6‐months starting at 2 months of age. Although a 2 and 4 months' duration of treatment did not show any significant difference with untreated animals, a 6 months treatment displayed reduced gene expression for most inflammation related genes.^18^

Pentosan polysulfate (PPS) is an FDA‐approved anti‐inflammatory and pro‐chondrogenic molecule, which has shown immunomodulation in MPS subtypes in preclinical^111^ and clinical settings.^112^ PPS was then tested in three cohorts of MPS IIIA mice injected weekly subcutaneously: 1‐week‐old mice treated at 25 mg/kg once weekly for 31 weeks (group 1); 5‐month‐old mice treated at 50 mg/kg for 12 weeks (group 2). Then, 5‐week‐old mice received continuous ICV PPS infusion for 11 weeks (60 mg/kg/day). Early SC PPS therapy showed reduced neuroinflammation and neurodegenerative features in MPS IIIA moue brain.^113^

Anakinra, an IL‐1 antagonist which inhibits IL‐1R, is an approved anti‐inflammatory drug. The Lundquist Institute, the Cure Sanfilippo Foundation and SOBI are sponsoring a phase II/III open‐label study (NCT04018755), which plans to enrol 20 children, aged 4 years old and older. After an initial 8 weeks observation period, patients will receive 100 mg of anakinra SC daily for 36 weeks.^74^, ^81^

### 4.4 Stop‐codon read‐through therapy

As a therapeutic strategy for nonsense mutations, several drugs have been used to produce a read‐through of premature stop codons. While aminoglycoside antibiotics including gentamicin and geneticin have been used widely for this purpose, there are additional molecules, which allows stop‐codon read‐through therapy. PTC124 (Ataluren) is a small molecule with no antibiotic properties or serious adverse events that can promote read‐through of disease‐causing premature termination codons without affecting stop codons at the end of coding sequences. These drugs were tested on MPS IIIB and MPS IIIC fibroblasts and no enzymatic response were observed with gentamicin, geneticin, and five other non‐aminoglycoside molecules (PTC124, RTC13, RTC14, BZ6, and BZ16) read‐through compounds. However, mRNA levels were nearly 2‐ and 1.5‐fold increased for MPS IIIB fibroblasts with G418 and MPS IIIC fibroblasts with RTC14 and PTC124, respectively.^114^

### 4.5 Inhibiting protein aggregation

Amyloidogenic protein aggregation is a common finding for many neurodegenerative diseases, including MPS III. CLR01, a molecular tweezer, which inhibits the self‐assembly of multiple amyloidogenic proteins. Then, 4.5‐month‐old MPS IIIA mice were treated with daily SC injection of CLR01 before massive amyloid aggregation occurs and over a period of 4.5 months. At 9‐month‐old, these mice showed a strong reduction in amyloid deposits and restoration of the autophagy‐lysosomal pathway associated with decreased neuroinflammation and better memory performance.^115^

### 4.6 Anti‐oxidant therapy

Coenzyme Q10 (CoQ10) levels are decreased in MPS IIIA fibroblasts compared to wild‐type controls. MPS IIIA and IIIB fibroblasts were supplemented with an antioxidant cocktail including CoQ10. Increased enzymatic activity was noticed in MPS IIIB but not in MPS IIIA fibroblasts. Decreased GAG storage was observed in some MPS IIIA and IIIB cell lines, especially the ones exhibiting enhanced exocytosis.^116^ This approach may be beneficial as an adjuvant therapy.^56^

## 5 IN VIVO ADENO‐ASSOCIATED VIRAL GENE THERAPY

Adeno‐associated viral (AAV) gene therapy enables in vivo transduction of the targeted cell types, which can be reached through vector delivery via various routes of administration (Figure 2). AAV gene therapy trials are summarised in Table 3.

**TABLE 3:** Gene therapy clinical trials for MPS III

Disease	Approach	Investigational medicinal product	Study status	Sponsor	Route of administration	Clinical trial identifier	Publications
MPS IIIA	AAV gene therapy	AAVrh.10‐SGSH‐IRES‐SUMF1 (SAF‐301)	Completed	Lysogene	Intraparenchymal	NCT01474343	103
		AAVrh.10‐h.SGSH (LYS‐SAF302)	Recruiting	Lysogene	Intraparenchymal	NCT03612869	
		scAAV9.U1a.hSGSH (ABO‐102)	Recruiting	Abeona Therapeutics	Intravenous	NCT02716246 NCT04088734	106
		AAVrh.9‐hSGSH (EGT‐101)	N/A	Esteve	ICV	N/A	135
	Lentiviral gene therapy	Autologous CD34+ cells transduced with LV.CD11b.hSGSH (OTL‐201)	Recruiting	University of Manchester Orchard Therapeutics	Intravenous	NCT04201405	124
MPS IIIB	AAV gene therapy	AAV5‐hNAGLU	Completed	UniQure Biopharma	IP	NCT03300453	114
		AAV9.CMV.hNAGLU (ABO‐101)	Recruiting	Abeona Therapeutics	Intravenous	NCT03315182	117

Abbreviations: AAV adeno‐associated virus; ICV, intracerebroventricular; IP, intraparenchymal; LV, lentivirus; MPS III, mucopolysaccharidosis type III; SUMF1, sulfatase modifying factor 1.

### 5.1 MPS IIIA

#### 5.1.1 Preclinical studies

Proof of concept of AAV gene therapy for MPS IIIA was initially reported in neonatal MPS IIIA mice after ICV injections of an AAV5 vector carrying both human SGSH and sulfatase modifying factor 1 (SUMF1) transgenes.^117^ SUMF1, which catalyses the post‐translational modification of a cysteine residue at the catalytic site to activate sulfatase enzymes, had been reported to have a synergistic effect on sulfatase activity when co‐expressed.^117^, ^118^ This showed a significant improvement of motor and cognitive behaviour, a reduction in lysosomal storage and neuroinflammatory markers.^117^ These results were reproduced in 2‐month‐old MPS IIIA mice IV injected with AAV1 and AAV8 vectors carrying murine SGSH targeting muscle and liver, respectively.^119^ A significant increase of plasma SGSH activity was associated with normalisation and decrease by half of GAG levels peripherally and in the CNS, respectively.^119^ Conversely AAV1 mediated gene therapy targeting the skeletal muscle could not demonstrate a significant therapeutic benefit.^119^ This designated the liver as an attractive therapeutic platform for production of secreted LSD enzymes, which will then be addressed to the CNS. Sorrentino et al developed a liver‐targeting AAV8 vector carrying a chimeric sulfamidase engineered by cloning the signal peptide from the highly secreted iduronate‐2‐sulfatase and the BBB‐binding domain (BD) from the Apolipoprotein B (ApoB‐BD) in the transgene cassette. IV injection in adult MPS IIIA mice showed increased plasmatic and cerebral activity, enhanced BBB transcytosis, and normalisation of the behavioural phenotype.^120^ AAV‐mediated liver‐directed gene therapy is expected to provide a transient effect in children. As AAV is considered as a non‐integrative vector, the vector copies will be progressively eliminated in parallel with the liver growth.^121^ The immune response triggered by the administration of AAV gene therapy generates neutralising antibodies cross‐reacting between various serotypes,^122^ which often precludes a safe and efficient re‐administration of AAV vector.

Intrastriatal injection of the neurotropic AAVrh10 capsid carrying human *hSGSH* and *hSUMF1* in presymptomatic adult MPS IIIA mice showed limited biodistribution around the injection site and local improvement of the neuropathology.^123^ Optimisation of the vector design and the route of administration favoured an ubiquitous promoter containing the chicken b‐actin promoter and a CMV enhancer (CAG) and a single depth injection method.^124^

IP administration of AAVrh.10‐CAG‐SGSH significantly reduced microgliosis and neuroinflammation in MPS IIIA mice and increased SGSH enzyme activity of at least 20% of the normal throughout the brain volume in large animals including dogs and Cynomolgus monkeys.^125^

AAV9 is a highly neurotropic AAV serotype, which has the ability to cross the BBB after systemic delivery and transduce the whole brain in animals^126^ and in humans.^127^ AAV9 vector encoding *hSGSH* under the control of the CAG promoter was systemically administered into 2‐month‐old MPS IIIA mice.^128^ Widespread gene expression was observed in both brain and liver. Increased survival, behavioural improvement, cerebral, and liver GAG clearance, remarkable reduction of neuroinflammation were sustainably reported.^128^ AAV gene therapy was assessed at different disease stages. Systemic injection of scAAV9‐U1a‐*hSGSH* in 1 to 3‐month‐old MPS IIIA mice displayed a remarkable improvement in learning ability at 7.5 months of age with a normal lifespan. However, injections in 6‐month‐old mice showed limited behavioural benefit at 7.5 months with mild improved lifespan. Injection in 9‐month‐old mice did not modify behaviour or survival despite a decrease in cerebral GAG.^129^ This study highlighted the importance of targeting early disease stages before irreversible neurological damage occurs. An alternative delivery strategy with IT administration of an AAV9 vector encoding SGSH was successfully performed in 2‐month‐old MPS IIIA mice.^130^ This interestingly reported as well prolonged survival, correction of the behavioural phenotype and neuropathology but an increased activity of the plasma SGSH activity and peripheral GAG clearance, highlighting a successful retrograde BBB crossing of the vector. This delivery strategy was validated in beagle dogs with ICM injection confirming transgenic expression throughout the CNS and liver and increased SGSH activity in CSF. High‐titre serum anti‐AAV9 antibodies only partially blocked CSF‐mediated gene transfer to the dog brain.^130^ Anti‐AAV antibody titres were reported lower in CSF than in serum samples of healthy and MPS IIIA children.^130^

#### 5.1.2 Clinical trials

A pilot phase I/II open‐label clinical trial (NCT01474343) sponsored by Lysogene was initiated in 2011. An AAVrh10 vector carrying *hSGSH* and *hSUMF1* transgenes (AAVrh.10‐SGSH‐IRES‐SUMF1) was injected intraparenchymally through a stereotaxic device with 12 needles in four MPS IIIA paediatric patients. Immunosuppressive treatment including mycophenolate mofetil and tacrolimus was initiated 15 days before surgery and maintained through the follow‐up with gradual dose reduction to inhibit the loss of transduced cells. The injected product was well tolerated without any adverse events related to it, furthermore, there was no increase in the number of infectious events, and no lack of compliance associated with immunosuppressive regimen. Although, the clinical efficacy endpoints including brain MRI, neurocognitive/behavioural tests and biological markers on blood, urine, and CSF were not met in all patients, the youngest patient displayed the highest improvement.^131^ An ongoing Lysogene‐sponsored open‐label, single‐arm phase II/III, clinical trial called AAVance (NCT03612869) aims to assess intracerebral administration of AAVrh10 encoding *hSGSH* only in 20 MPS IIIA patients older than 6 months with a DQ >50. Change from baseline in DQ is the primary outcome of the study. The first patient was injected in February 2019 and 19 patients out of 20 have been recruited in June 2020. The trial is currently on hold following localised findings on brain MRIs suggested to be associated with delivery.^132^

Building upon AAV9 preclinical data, Abeona Therapeutics is sponsoring two recruiting phase I/II open‐label clinical trials to assess the safety and efficacy of a single intravenous injection of scAAV9‐U1a‐*hSGSH*.^133^ Then, 22 young higher‐functioning MPS IIIA patients either aged 6 months to 2 years or older than 2 years with DQ ≥60 are recruited. Dose escalation entails three groups from low dose (0.5 × 10e13 vg/kg), mid‐dose (1 × 10e3 vg/kg) to high dose (3 × 10e13 vg/kg). Preliminary data at 24, 12, and 6 months post‐injection from the three dose‐escalating groups, respectively, highlighted a provisional acceptable safety in all patients with significant time‐ and dose‐dependent reduction of HS levels and liver volume, and stabilisation or improvement of adaptive behaviour and/or cognitive function.^134^ Study ABT‐003 (NCT04088734) is another clinical trial enrolling 12 MPS IIIA patients with a DQ lower than 60 in middle and advanced phases of the disease. Recruited patients will receive a single dose corresponding to the high dose of the Transpher A study, that is, 3 × 10e13 vg/kg.

An AAV9 vector encoding human sulfamidase (SGSH) gene administered via a single ICV injection is currently being assessed in a phase I/II clinical trial sponsored by Esteve.^135^

### 5.2 MPS IIIB

#### 5.2.1 Preclinical studies

The Muenzer lab demonstrated the proof of concept of AAV gene therapy for MPS IIIB in the early 2000s. IP injections per adult MPS IIIB mouse of an AAV2 vector encoding the human α‐N‐acetylglucosaminidase (NAGLU) cDNA under the transcriptional control of the neuronal promoter NSE (AAV2.NSE.hNAGLU) showed long‐term expression and local improvement of the neuropathology around the injection site up to 1.5 mm.^136^ A similar vector using the ubiquitous promoter CMV (AAV2.CMV.hNAGLU) was used for combined IV and ICM administrations following an IV infusion of mannitol to maximise cerebral delivery in adult MPS IIIB mice. This showed a mean increased lifespan of 6 months, systemic and cerebral long‐term transgene expression associated with improved clearance of storage materials.^137^ The neurotropic AAV5 capsid was used to design an AAV5.CAG.hNAGLU vector, which was administered by IP injections in neonatal MPS IIIB mice. This showed improvement of lifespan, motor function and neuropathology.^43^ [Correction added on xx December 2020 after its first online publication: The preceding sentence has been updated to include the effect on life span.] Subsequently, a triple‐capsid mutant (tcm) modification of AAV8 developed by the University of Florida and Lacerta Therapeutics was used to increase the transduction of the MPS IIIB mouse brain administered either via six IP or ICM injections enabling supraphysiological cerebral NAGLU expression.^138^ The broad transduction enabled by the neurotropic AAV5 capsid was further exploited using a recombinant AAV5 vector encoding hNAGLU cDNA under the control of the murine phosphoglycerate kinase promoter (AAV5.PGK.hNAGLU).^139^ Then, 5 × 10e11vg/kg per animal was delivered to MPS IIIB dogs through eight IP injections. The tolerance was considered satisfactory. NAGLU activity was increased in most parts of the brains except the most rostral and caudal regions with improved neuropathology markers. Immunosuppression was deemed necessary to prevent vector‐triggered neuroinflammation and clearance of the transduced cells. The immune response suspected to be directed both against the capsid and the transgene as observed in MPS I^140^ was suppressed with a combination of mycophenolate mofetil and cyclosporine.^139^

The neurotropic AAV9 capsid was used to design a vector AAV9.CMV.hNAGLU administered IV in adult MPS IIIB mice with high doses (up to 2 × 10e13vg/kg). Long‐term effect over 18 months was observed with increased survival, improved motor function, cerebral and systemic supraphysiological levels of NAGLU activity, clearance of storage materials in various systemic organs and the CNS, correction of astrocytosis, and neurodegeneration.^141^ This vector was administered at same dose in non‐human primates enhancing no detectable toxicity and global cerebral and systemic transduction.^142^ Of note, systemic pre‐existing anti‐AAV9 antibodies did not affect brain transduction.^142^ An approach using the intraparenchymal route for injection of various AAV capsid AAV5, 8, 9, rh10 in MPS IIIB mice identified the AAV8 capsid as providing the highest transduction and best suitable capsid candidate for translation.^143^ [Correction added on xx December 2020 after its first online publication: The preceding sentence has been update to reflect the correct approach.]

#### 5.2.2 Clinical trials

Uniqure Biopharma and the Institut Pasteur led an open‐label phase 1/2 clinical trial (NCT03300453) to assess the safety and efficacy of an AAV5 vector encoding hNAGLU. The vector was administered IP in 16 sites across the brain through eight burr holes for a total dose of 4 × 10e12vg. A concomitant immunosuppression based on tacrolimus and mycophenolate mofetil was started 14 days before gene therapy and maintained throughout follow‐up. Four patients aged 20, 26, 30, and 53 months were recruited between 2012 and 2014. At 30 months post‐injection, the product and procedure of administration were well tolerated. Neurocognitive progression of the disease was improved in all patients compared to natural history with a better outcome in the youngest patient treated. Undetectable CSF NAGLU activity before injection showed persisting increase to 15% to 20% of unaffected children. CSF HS levels were not modified. Neutralising antiAAV5 antibodies were not detected at inclusion and during follow‐up.^144^ As in a similar MPS IIIA trial, the potential beneficial effect of chronic immunosuppression on neuroinflammation, a pathophysiological landmark of the MPS IIIB neurological disease, cannot be ruled out.^145^

An ongoing phase 1/2 clinical trial (NCT03315182) Transpher B, assessing the safety and efficacy of ABO‐101, a self‐complementary AAV9 vector encoding hNAGLU (AAV9.CMV.hNAGLU) delivered with a single IV injection is sponsored by Abeona Therapeutics. This non‐randomised, open‐label, dose‐escalation study is recruiting 12 patients. Eight patients have been enrolled so far in low (2 × 10e13vg/kg; n = 2), medium (5 × 10e13vg/kg; n = 5) and high dose cohorts (1 × 10e14vg/kg; n = 1).^146^ Two and four patients enrolled in the low and medium dose cohorts, respectively, have shown a good safety after a median follow‐up of 15 and 3 months, respectively. Sustained reduction in CSF and urine HS levels, urine GAGs and liver volume were observed up to 18 months follow‐up.^147^

### 5.3 MPS IIIC

An AAV gene therapy approach using the novel capsid variant AAV‐TT was successfully tested in a MPS IIIC mouse model. This mouse model shows hyperactivity from 6 months of age, cognitive decline from 10 months and ataxic gait from 12 months with cerebral and systemic accumulation of HS.^148^ The codon‐optimised human cDNA of the deficient transmembrane lysosomal HS acetyl‐CoA:α‐glucosaminide N‐acetyltransferase (HGSNAT) protein was cloned under the transcriptional control of the CAG promoter (AAV‐TT.CAG.hcoHGSNAT). Two‐month‐old MPS IIIC mice received striatal injection with 5.2 × 10e9vg/mouse. AAV‐TT was compared to benchmark neurotropic AAV9 capsid. AAV‐TT treated animals showed an improved correction of the pathological behaviour, increased clearance of storage materials, and correction of neuroinflammation in a higher number of brain regions compared to AAV9.^149^ This preclinical programme is developed by Phoenix Nest.^150^

## 6 EX VIVO LENTIVIRAL GENE THERAPY

Lentiviral gene therapy requires collection of HSCT from a donor (heterologous procedure) or the patient (homologous procedure), which will be transduced ex vivo via the desired lentiviral vector. The patient will undergo myeloablative conditioning before reinjection of transduced HSCT (Figure 2). Lentiviral gene therapy trials are summarised in Table 3.

### 6.1 MPS IIIA

Ex vivo lentiviral (LV) mediated gene therapy for MPS IIIA has been developed in the early 2010s by the Bigger lab in Manchester, UK. A first LV vector encoding the hSGSH cDNA under the control of the viral promoter spleen focus forming virus SFFV was used to transduce either murine WT or MPS IIIA haematopoietic stem cells (HSC) vs WT HSC transplantation without gene therapy. All approaches showed an improved neurological disease with better correction achieved with LV‐transduced WT HSC. In this treated group, 10% of WT cerebral SGSH activity was observed with increased mean survival by 17 weeks (66 vs 49 weeks in WT HSC treated vs untreated), normalisation of hyperactivity phenotype, near normalisation of cerebral HS and GM2 gangliosides levels and improved neuropathology.^40^ Vector optimisation tested monocyte/microglia specificity of mammalian promoters driving the expression of human codon‐optimised SGSH gene. The CD11b promoter displayed a significant monocyte/B‐cell specificity driving higher cerebral SGSH expression after LV‐HSC gene therapy in MPS IIIA mice. This corrected the hyperactivity phenotype, neuroinflammation and normalised cerebral HS and GM2 gangliosides levels.^151^ Pre‐clinical safety and efficacy studies of a GMP‐graded CD11b.hSGSH LV vector validated scale‐up manufacturing and cryopreservation. No vector shedding and transmission to germline was observed. The genotoxicity risk was considered low and similar to other LV vectors used for clinical applications.^152^

Induced pluripotent stem cells (iPSC)‐derived neural stem cells reprogrammed from mouse embryonic fibroblasts were transduced with a LV vector encoding a transgene cassette containing hNAGLU and the receptor‐binding motif of IGF2 as previously tested.^78^ The transduced iPSC neural stem cells were injected via ICV or IP routes in neonatal MPS IIIB mice. This showed a sustained engraftment at a 9‐month timepoint associated with local improvement of neuropathology decreased with evidence of neighbouring cross‐correction in adjacent brain areas.^153^

LV gene therapy is currently developed in collaboration between Manchester University and Orchard Therapeutics. A phase 1/2 open‐label clinical trial (NCT04201405) is recruiting up to five MPS IIIA patients aged 3 to 24 months to assess the safety and efficacy of OTL‐201. A patient treated before this trial on a “specials” licence received a busulfan‐mediated myelo‐ablative conditioning followed by HSC transplantation by autologous CD34+ cells transduced with LV.CD11b.hSGSH vector. At 12 months post‐transplantation, evidence of sustained engraftment was achieved with leucocyte enzymatic levels achieving 25 times higher levels than the upper normal value and a rapid decrease of HS in CSF, blood, and urine.^154^

### 6.2 MPS IIIB

In vitro proof of concept of LV encoding hNAGLU cDNA showed a 20‐fold increase of enzymatic levels for 2 months in MPS IIIB fibroblasts, normalising GAG levels.^155^ in vivo gene therapy with single IV injection of LV.CMV.hNAGLU in adult MPS IIIB mice showed a 6 months sustained expression of NAGLU in various peripheral organs especially liver and spleen with appropriate decrease of visceral GAG content.^156^ A single neonatal IV injection of LV.MND.hNAGLU in MPS IIIB mice confirmed a good correction of the systemic phenotype but a mild improvement of survival and motor function.^157^

As for MPS IIIA, a similar ex vivo approach is being developed for MPS IIIB. Proof of concept of ex vivo LV gene therapy encoding NAGLU under the control of the myeloid‐specific promoter CD11b transducing HSC (LV.CD11b.hNAGLU) showed long‐term supraphysiological enzyme activity in leucocytes, brain and liver, normalisation of systemic and cerebral HS levels, increased survival, normalisation of hyperactivity behaviour and neuroinflammation.^19^ A preclinical collaborative programme between Manchester University and Orchard Therapeutics is now developing a clinical grade LV.CD11b.hNAGLU vector as an investigational medicinal product OTL‐202.

## 7 EXPERIMENTAL THERAPIES INCLUDING GENE EDITING

RNA‐based therapies and gene editing tools, such as Clustered Regularly Interspaced Short Palindromic Repeats (CRISPR)/(CRISPR‐associated system) Cas9, are enabling novel approaches to treat inherited diseases. Besides the off‐target effects, these therapies work well in vitro and have shown success in rodent models although concerns for translation have been highlighted including off‐target effects, immunisation against the editing tool and limited biodistribution, especially for neurons.^158^, ^159^

### 7.1 Combined gene therapy approaches

An experimental design in neonatal MPS IIIB mice has compared IP injection of AAV5‐NAGLU (IC‐AAV), IV injection of LV‐NAGLU (IV‐LENTI) or the combination of both. Although all treatment groups had some increased improvement in survival, neuropathology, motor and hearing functions, the combined treatment group displayed the best outcome with an increased survival by 90%, 71%, and 32% compared to untreated, IV‐LENTI only and IC‐AAV only, respectively.^157^ This work highlighted the synergistic effect of therapies targeting both the liver and the brain, although a combination if two different viral vectors remain up to now an obstacle to safe translation.

### 7.2 RNA‐based therapies

To minimise uGAG production, small interfering RNAs (siRNAs) were successfully used in MPS IIIA fibroblasts to reduce mRNA expression of four genes (*XYLT1*, *XYLT2*, *GALTI*, and *GALTII)* whose products are involved in GAG synthesis.^160^ Similarly short hairpin RNA silencing *EXTL2* and *EXTL3* genes showed reduction of GAG synthesis and HS clearance in MPS IIIA fibroblasts.^161^ siRNAs targeting *EXTL2* and *EXTL3* were also effective in MPS IIIC fibroblasts.^162^

To rescue normal RNA processing in cells with mutations affecting splicing, specifically modified U1 small nuclear RNAs (snRNAs) were successfully tested in three fibroblast lines of MPS IIIC patients with mutations affecting the donor splicing site of *HGSNAT* gene (c.234 + 1G > A, c.633 + 1G > A and c.1542 + 4dupA). Partial correction of gene expression and enzyme activity was obtained in the c.234 + 1G > line, suggesting that this approach may be an option for some splice‐site mutations.^110^

### 7.3 CRISPR/Cas9 and base editing strategies

Most recent genome‐editing tools such as zinc finger nucleases (ZFNs), transcription activator‐like effector nucleases (TALENs), clustered regularly interspaced short palindromic repeat (CRISPR)‐Cas9 system, and lately described CRISPR‐Cas9‐based editors and prime editing^163^ have shown the ability to create double stranded DNA break (DSB) at a specific gene locus which can then be repaired either by insertion of a sequence of interest thanks to flanking homologous DNA arms (ZFN, TALEN) or by homology directed repair for CRISPR‐derived technologies.^164^, ^165^ A novel innovative strategy for genome editing is CRISPR‐mediated base editing which does not depend on the repair of DSB. Base editing technology utilises an inactive (ie, non‐cutting) Cas9 enzyme or Cas9 nickase (cutting only one of the two DNA strands) to recruit base‐modifying enzymes, such as cytosine deaminase or adenosine deaminase to specific locations in the genome.^166^, ^167^ Adenine and cytidine deaminases convert C‐G to T‐A base pairs, or vice versa. Thus, this genome‐editing tool is mutation‐specific and limited to pathogenic variants involving C or A residues in the domain of the protospacer adjacent motif, the specific sequence used by Cas9 for precise binding to the host genome.^163^ Prime editing, is another editing strategy, which does not require DSB. A reverse transcriptase is fused to a Cas9 nickase and a prime editing guide RNA (pegRNA) to correct all transversion mutations (C → A, C → G, G → C, G → T, A → C, A → T, T → A, and T → G) as well as targeted deletions and insertions. It is more effective, accurate, and less genotoxic.^168^

MPS III subtypes have targetable mutations for base editing. For example, while p.R245H (CGC → CAC) in the *SGSH* gene, p. E153K (GAG → AAG) in the *NAGLU* gene, p.R351X in the *HGSNAT* gene has the potential to be corrected through ABEmax base editors, p.R355X in the *GNS* gene can be a correction template for ABEmax‐Cpf1 fusion (TTTV) base editor. Although both base editors and CRISPR/Cas9 share the advantage of high precision in DNA targeting,^169^ base editors show less off‐target effects and are independent of cellular repair systems, homology directed repair and non‐homologous end joining, in comparison to its CRISPR/Cas9 counterpart.

## 8 CONCLUSION

MPS III is one of the most common subtypes of MPS. Well‐defined natural history is characterised by predominant neurodegeneration contrasting with mild somatic features. Although some other MPS subtypes can benefit of approved therapeutics with ERT (MPS I, II, IVA, VI, and VII) and HSCT (MPS I) targeting systemic and CNS symptoms respectively, no disease‐changing therapy is yet approved for MPS III. Supported by promising preclinical studies, the ongoing clinical trials of ERT and particularly gene therapy are generating hope and scrutiny from the whole community of patients and families, physicians and clinical teams, scientists and investors. In parallel, novel research strategies based on gene editing are being explored to develop mutation‐specific and personalised medicines to these vulnerable patients. The main obstacles faced to develop a curative therapy are effective BBB crossing, diffuse cerebral biodistribution, sustained effect with adequate dosing and early diagnosis. In MPS III, the early initiation of any effective therapy remains the cornerstone of successful management to prevent irremediable neuronal loss. It is expected that a novel disease‐modifying therapy would profoundly modify the pathway of diagnosis and management of these patients, promoting potential access to newborn screening.

## CONFLICT OF INTEREST

B. S. Y. and J. B. have no conflict of interest. S. A. J. is an investigator and consultant for Shire/Takeda; consultant for Biomarin; investigator and consultant for Alexion; investigator, consultant, SAB member and Stockholder for Orchard. J. D. is an investigator for Sanofi Genzyme; consultant for Biomarin and Sanofi Genzyme; received educational grants from Shire and Biomarin.
